# Crystal structure, Hirshfeld surface and frontier mol­ecular orbital analysis of 10-benzyl-9-(4-hydroxy-3-meth­oxy­phen­yl)-3,3,6,6-tetra­methyl-3,4,6,7,9,10-hexa­hydro­acridine-1,8(2*H*,5*H*)-dione

**DOI:** 10.1107/S2056989022006557

**Published:** 2022-07-14

**Authors:** V. Sughanya, B. Loganathan, D. Praveenkumar, J. Ayyappan, M. L. Sundararajan, A. Prabhakaran, A. Dhandapani, N. Suresh Babu

**Affiliations:** a Department of Chemistry, Periyar Government Arts College, Cuddalore-607 001, Tamil Nadu, India; b Department of Chemistry (Science and Humanities), Dr. N.G.P. Institute of Technology, Coimbatore-641 048, Tamil Nadu, India; c Department of Chemistry, Swami Vivekananda Arts and Science College, Orathur-605 601, Tamil Nadu, India; d Department of Physics, Government College of Engineering-Sengipatti, Thanjavur-613 402, Tamil Nadu, India; e Department of Chemistry, Annamalai University, Annamalai Nagar-608 002, Tamil Nadu, India; f Department of Chemistry, CK College of Engineering and Technology, Chellangkuppam, Cuddalore-607003, Tamil Nadu, India; g Department of Chemistry, Government College of Engineering-Sengipatti, Thanjavur-613 402, Tamil Nadu, India; Moscow State University, Russia

**Keywords:** crystal structure, benzyl­amine, acridinedione, hydrogen bonding, Hirshfeld analysis, frontier orbitals

## Abstract

In the fused ring system of the title mol­ecule, C_31_H_35_NO_4_, the conformation of the central di­hydro­pyridine ring is inter­mediate between boat and envelope with the N and the opposite C atoms lying out of the basal plane. The conformations of terminal rings are close to envelope, with the atoms substituted by two methyl groups as the flaps. In the crystal, the mol­ecules are linked by O—H⋯O hydrogen bonds into helical chains. The Hirshfeld surface analysis indicates that the most important contributions to the crystal packing are from H⋯H (63.2%), O⋯H/H⋯O (20.1%) and C⋯H/H⋯C (14.4%) contacts. Quantum chemical calculations of the frontier mol­ecular orbitals were carried out to characterize the chemical reactivity of the title compound.

## Abstract 2

In the acridinedione core of the title mol­ecule, C_31_H_35_N_1_O_4_, the central ring adopts a flattened-boat conformation, whereas the cyclo­hexenone rings adopt envelope conformations.

## 1. Chemical context

The acridine fragment is a part of a number of naturally occurring substances, and its derivatives have been used as photoinitiators. Acridine-1,8-diones have been shown to have very high lasing efficiencies and have been used as dyes (Niknam & Damya, 2009 ▸). Some acridine derivatives (Nasim & Brychcy, 1979 ▸; Thull & Testa, 1994 ▸), also well known as therapeutic agents, have a wide range of applications in the pharmaceutical and dye industries. These include compounds that are used as anti-cancer (Sondhi *et al.*, 2004 ▸; Sugaya *et al.*, 1994 ▸; Kimura *et al.*, 1993 ▸), anti-tubercular (Aly & Abadi, 2004 ▸; Tripathi *et al.*, 2006 ▸), anti-inflammatory (Chen *et al.*, 2002 ▸), anti-malarial (Kumar *et al.*, 2009 ▸; Tomar *et al.*, 2010 ▸), anti-viral (Gupta & Jaiswal, 2010 ▸; Tonelli *et al.*, 2011 ▸), anti-parasitic (Di Giorgio, *et al.*, 2005 ▸) and fungicidal agents (Srivastava & Nizamuddin, 2004 ▸). In this context, we report here the synthesis, crystal structure, Hirshfeld surface and frontier mol­ecular orbital analysis of the title acridine-1,8-dione derivative.

## 2. Structural commentary

**Figure 1:**
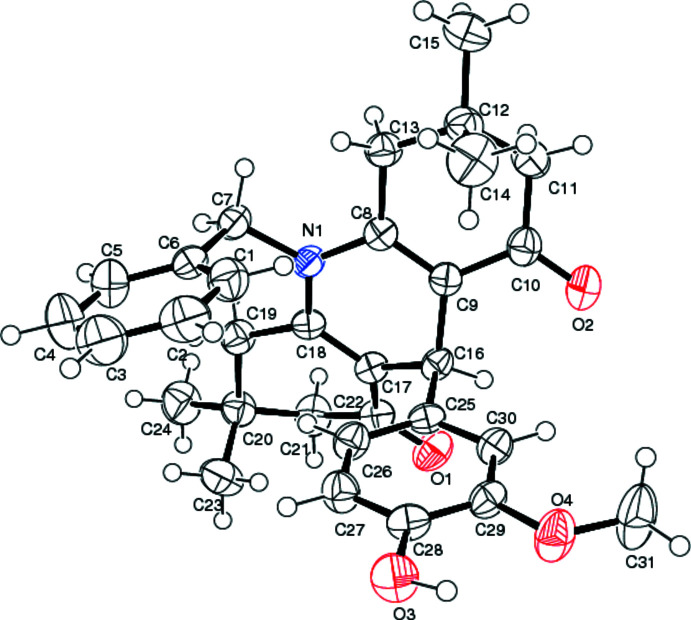
The mol­ecular structure of the title compound. Displacement ellipsoids are drawn at the 50% probability level.

The title compound (Fig. 1 ▸) crystallizes in the monoclinic space group *P*2_1_/*n* with *Z* = 4. The conformation of the central di­hydro­pyridine ring is inter­mediate between boat and envelope: four atoms (C8, C9, C17 and C18) form the basal plane with a deviation of 0.008 (2) Å for all of them, whereas atoms N1 and C16 deviate from this plane by 0.168 (2) and 0.476 (2) Å, respectively. The conformations of the terminal C8–C13 and C17–C22 rings are close to envelope with C12 and C20, respectively, as the flap atoms. The basal planes of these envelopes are twisted, and the deviations of corresponding atoms from their least-squares planes are between 0.005 (2) and 0.100 (2) Å. The N1 atom has an essentially planar environment, deviating from the plane through atoms C7, C8 and C18 by only 0.018 (2) Å. The bond lengths in the N1—C8—C9—C10—O2 and N1—C18—C17—C22—O chains indicate π-conjugation of N1 with the carbonyl groups C10=O2 and C22=O1 (Table 1 ▸). All other bond lengths and angles in the title structure are within the ranges normal for analogous compounds (Allen *et al.*, 1987 ▸; Thamotharan *et al.*, 2021 ▸; Allah *et al.*, 2021 ▸; Mohamed *et al.*, 2013 ▸; Akkurt *et al.*, 2014 ▸).

## 3. Supra­molecular features and Hirshfeld analysis

**Figure 2:**
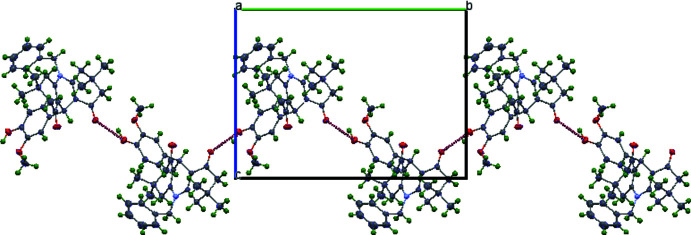
Packing view of the title compound showing the O—H⋯O inter­molecular hydrogen bonds.

In the crystal, the mol­ecules are linked *via* O3—H3*A*⋯O1^i^ hydrogen bonds [symmetry code (i): −*x* + 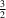 , *y* − 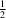 , −*z* + 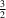 ] forming helical chains along the *b*-axis direction (Fig. 2 ▸, Table 2 ▸). The chains are further connected by weak C7—H7*B*⋯O1^ii^ hydrogen bonds [symmetry code (ii): *x* − 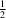 , −*y* + 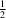 , *z* − 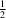 ] forming sheets parallel to (10 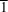 ).

**Figure 3:**
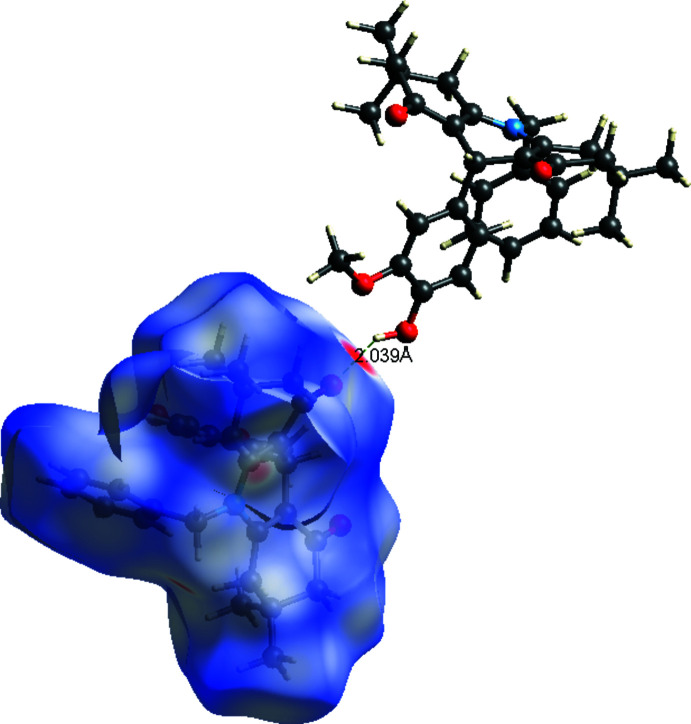
View of the three-dimensional Hirshfeld surface of the title mol­ecule plotted over *d*_norm_ in the range −0.436 to 1.583 a.u.

**Figure 4:**
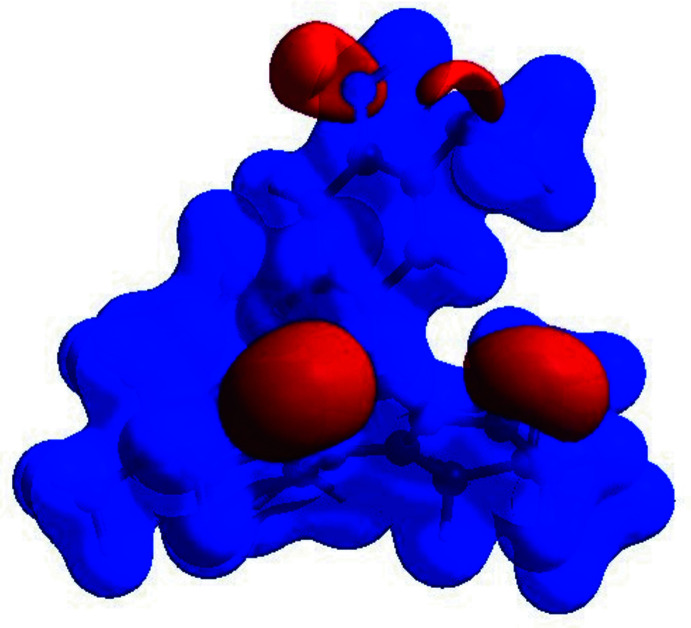
View of the three-dimensional Hirshfeld surface of the title mol­ecule plotted over electrostatic potential energy in the range −0.0500 to 0.0500 a.u. calculated with the STO-3 G basis set at the Hartree–Fock level of theory. The hydrogen-bond donating and acceptor areas are viewed as blue and red regions, respectively, around atoms, corresponding to positive and negative potentials.

**Figure 5:**
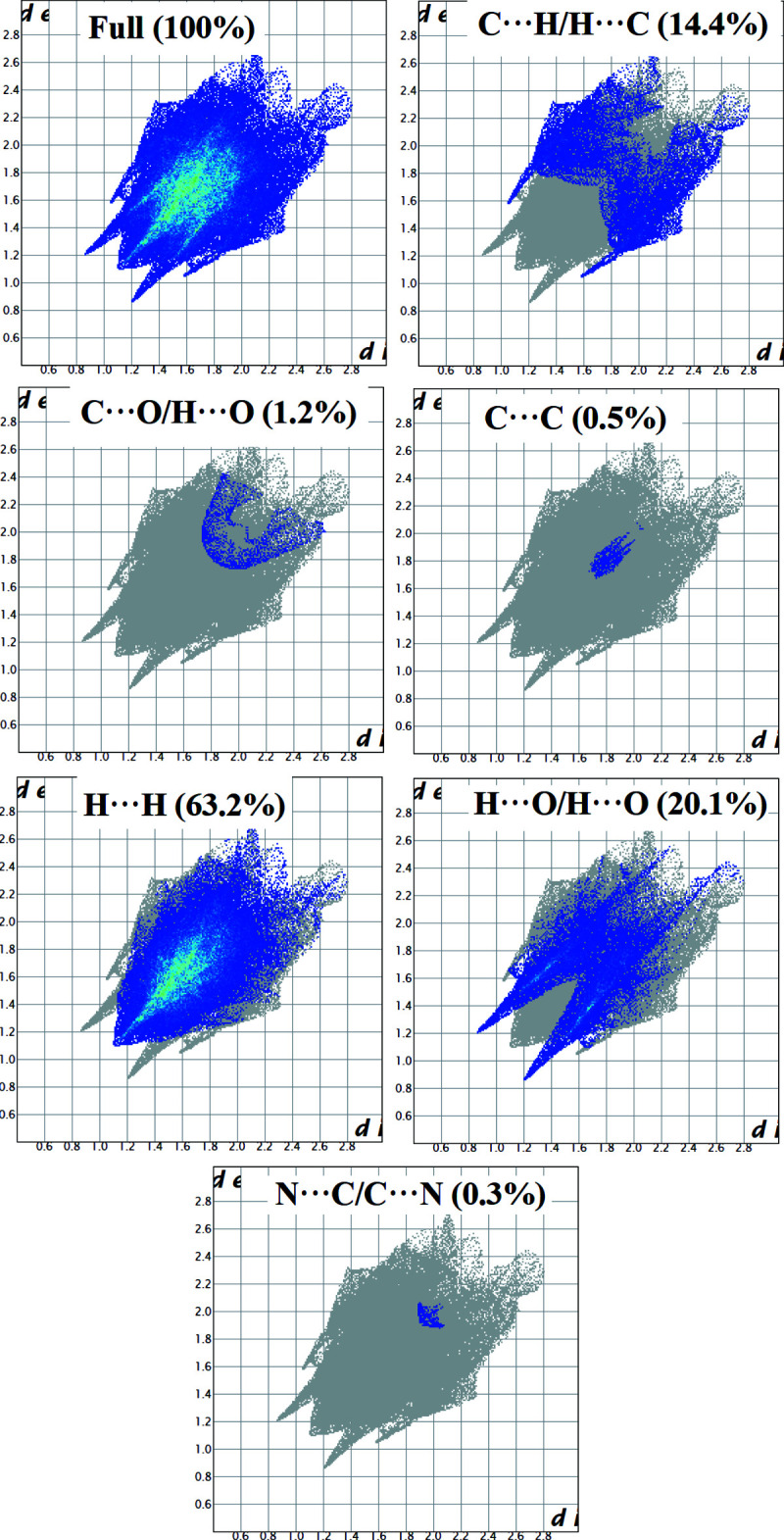
The two-dimensional fingerprint plot showing all inter­actions and those delineated into C⋯H/H⋯C, C⋯O/O⋯H, C⋯C, H⋯H, O⋯H/H⋯O and N⋯C/C⋯N contacts.

To qu­antify the inter­molecular contacts in the crystal, Hirshfeld surfaces and two-dimensional fingerprint plots were generated using *Crystal Explorer* (Version 17.5; Turner *et al.*, 2017 ▸). The Hirshfeld surface mapped over *d*_norm_ in the range −0.436 to 1.583 a.u. (Fig. 3 ▸) show the inter­molecular contacts as red-coloured spots, which indicate the C—H⋯O and O—H⋯O hydrogen bonds. The red and blue regions corresponding to negative (hydrogen-bond acceptors) and positive (hydrogen-bond donors) potentials on the Hirshfeld surface mapped over electrostatic potential are shown in Fig. 4 ▸. The two-dimensional fingerprint plots are presented in Fig. 5 ▸. The H⋯H contacts comprise 63.2% of the total inter­actions. Besides these contacts, O⋯H/H⋯O (20.1%) and C⋯H/H⋯C (14.4%) inter­actions make significant contributions to the total Hirshfeld surface. The percentage contributions of the N⋯C/C⋯N, C⋯O/H⋯O, and C⋯C contacts are 0.3, 1.2 and 0.5%, respectively.

## 4. Frontier mol­ecular orbital analysis

**Figure 6:**
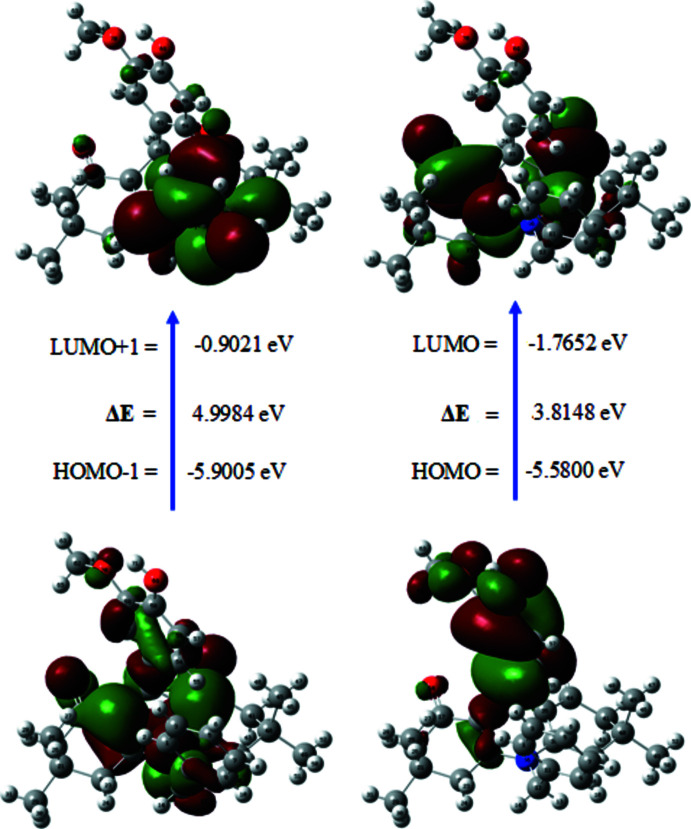
The frontier mol­ecular orbitals of the title mol­ecule.

The chemical reactivity of the title compound was studied by frontier mol­ecular orbital analysis. For the calculation, the mol­ecular structure obtained from X-ray diffraction data was used as the mol­ecular model. The energy levels, summarized in Table 3 ▸, were computed at the DFT-B3LYP/6-311G++(d,p) level of theory as implemented in *Gaussian09W* (Frisch *et al.*, 2013 ▸). The calculated frontier mol­ecular orbitals, LUMO+1, LUMO, HOMO, and HOMO-1, are shown in Fig. 6 ▸. The energies of LUMO+1, LUMO, HOMO and HOMO−1 were calculated to be −0.9021, −1.7652, −5.5800 and −5.9005 eV, respectively, and the energies required to excite one electron from HOMO−1 to LUMO+1 and from HOMO to LUMO are 4.9984 and 3.8148 eV, respectively. The chemical hardness, chemical potential, chemical softness and electrophilicity index of the title mol­ecule are listed in Table 4 ▸. The electrophilicity index value of 3.3429 eV shows the global electrophilic nature of the mol­ecule. Based on the wide band gap and chemical hardness value of 2.0174 eV, the title mol­ecule seems to be hard.

## 5. Database survey

A search of the Cambridge Structural Database (CSD, Version 5.43, updated September 2021; Groom *et al.*, 2016 ▸) for the acridine-1,8(2*H*)dione unit resulted in 22 hits. They include the following acridine-1,8(2*H*)dione derivatives similar to the title compound: 4-eth­oxy­phenyl (QEDYAB; Sughanya & Sureshbabu, 2012 ▸), 3,4-di­meth­oxy­phenyl (PUSJEU; Sureshbabu & Sughanya, 2015 ▸) and 3-eth­oxy-4-hy­droxy­phenyl (MULWUO; Suresh Babu *et al.*, 2020 ▸). In the title compound, the dihedral angle between the phenyl and di­hydro­pyridine rings is 85.39 (2)°, similar to the values observed for the 4-eth­oxy­phenyl analogue QEDYAB, the 3,4-di­meth­oxy­phenyl analogue PUSJEU, and 3-eth­oxy-4-hy­droxy­phenyl analogue MULWUO, for which the dihedral angles are 75.20 (4), 89.47 (9) and 85.81 (2)°, respectively.

## 6. Synthesis and crystallization

A mixture of benzyl­amine (0.214g, 2 mmol), 4-hy­droxy-3-meth­oxy­benzaldehyde (0.304g, 2 mmol) and 5,5-di­methyl­cyclo­hexane-1,3-dione (0.56g, 4 mmol) was dissolved in 25 ml of acetic acid. The solution was refluxed for 2 h with the reaction being monitored by TLC. After the reaction was about to the end, the reaction mixture was poured into 150 ml of ice-cold water, stirred at 298–303K for 10 min and then kept at room temperature for 12 h. The solid was filtered, washed repeatedly with water and dried. Yellow single crystals suitable for X-ray diffraction were obtained from 95% ethanol (m.p. 483 K, 0.718 g, 1.48 mmol, yield 74%). IR (KBr): cm^−1^ 2957-2871, 1634, 1455, 1373. ^1^H NMR(400 MHz, CDCl_3_): 0.90 (*s*, 6H), 0.99 (*s*, 6H), 2.21 (*s*, 4H), 2.40 (*dd*, 4H), 3.86 (*s*, 3H), 4.90 (*s*, 2H), 5.24 (*s*, 1H), 5.51 (*s*, 1H), 6.56 (*d*, 1H), 6.70 (*d*, 1H), 7.12 (*d*, 1H), 7.17 (*s*, 2H), 7.35–7.40 (*m*, 3H). ^13^C NMR (100 MHz, CDCl_3_): 28.11, 28.65, 31.70, 32.73, 40.27, 48.73, 50.05, 55.88, 111.90, 113.60, 115.44, 119.45, 125.38, 128.01, 129.25, 137.10, 138.36, 143.69, 145.92, 150.31, 195.90. ESI–MS: *m*/*z*:485.12 [*M* + H]^+^.

## 7. Refinement

Crystal data, data collection and structure refinement details are summarized in Table 5 ▸. Hydrogen atoms were fixed geometrically and treated as riding atoms, with C—H = 0.93–0.97 Å and *U*_iso_(H) = 1.2*U*_eq_(C) or 1.5*U*_eq_(C-meth­yl).

## Supplementary Material

Crystal structure: contains datablock(s) I, global. DOI: 10.1107/S2056989022006557/yk2171sup1.cif

Structure factors: contains datablock(s) I. DOI: 10.1107/S2056989022006557/yk2171Isup2.hkl

Supporting information file. DOI: 10.1107/S2056989022006557/yk2171Isup3.cml

CCDC reference: 2153580

Additional supporting information:  crystallographic information; 3D view; checkCIF report

## Figures and Tables

**Table 1:** Selected bond lengths (Å)

C8—C9	1.365 (3)	C17—C18	1.367 (3)
C8—N1	1.404 (3)	C17—C22	1.459 (3)
C9—C10	1.462 (3)	C18—N1	1.400 (3)
C10—O2	1.236 (3)	C22—O1	1.240 (3)

**Table 2:** Hydrogen-bond geometry (Å, °)

*D*—H⋯*A*	*D*—H	H⋯*A*	*D*⋯*A*	*D*—H⋯*A*
O3—H3*A*⋯O1^i^	0.94 (4)	2.07 (4)	2.780 (2)	131 (3)
C7—H7*B*⋯O1^ii^	0.97	2.41	3.260 (3)	146

Symmetry codes: (i) 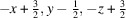 ; (ii) 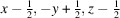 .

**Table 3:** The frontier mol­ecular orbital energies of title compound

Orbitals	a.u	eV	Type
*V*_136_	−0.00997	−0.27129	LUMO+5
*V*_135_	−0.02093	−0.56953	LUMO+4
*V*_134_	−0.02288	−0.62260	LUMO+3
*V*_133_	−0.02951	−0.80301	LUMO+2
*V*_132_	−0.03315	−0.90205	LUMO+1
*V*_131_	−0.06487	−1.76519	LUMO
*O*_130_	−0.20506	−5.57995	HOMO
*O*_129_	−0.21684	−5.90050	HOMO−1
*O*_128_	−0.23178	−6.30704	HOMO−2
*O*_127_	−0.23655	−6.43684	HOMO−3
*O*_126_	−0.24414	−6.64337	HOMO−4
*O*_125_	−0.26023	−7.08120	HOMO−5

**Table 4:** The global reactivity descriptors of the title compound (eV)

Frontier mol­ecular orbitals	Energy
*E*_HOMO_	−5.5800
*E*_LUMO_	−1.7652
*E*_HOMO-1_	−5.9005
*E*_LUMO+1_	−0.9021
(*E*_HOMO_–*E*_LUMO_) gap	3.8148
(*E*_HOMO-1_00*E*_LUMO+1_) gap	4.9984
Chemical potential (μ)	3.6726
Chemical hardness (η)	2.0017
Chemical softness (*S*)	0.4957
Electrophilicity index (ω)	3.3429

**Table 5:** Experimental details

Crystal data	
Chemical formula	C_31_H_35_NO_4_
*M*_r_	485.60
Crystal system, space group	Monoclinic, *P*2_1_/*n*
Temperature (K)	296
*a*, *b*, *c* (Å)	10.4430 (6), 18.4563 (11), 14.2378 (9)
β (°)	107.930 (2)
*V* (Å^3^)	2610.9 (3)
*Z*	4
Radiation type	Mo *K*α
μ (mm^−1^)	0.08
Crystal size (mm)	0.40 × 0.30 × 0.20

Data collection	
Diffractometer	Bruker Kappa APEXII
Absorption correction	Multi-scan (*SADABS*; Bruker, 2016 ▸)
*T*_min_, *T*_max_	0.953, 0.982
No. of measured, independent and observed [*I* > 2σ(*I*)] reflections	37271, 5135, 3061
*R*_int_	0.096
(sin θ/λ)_max_ (Å^−1^)	0.617

Refinement	
*R*[*F* ^2^ > 2σ(*F* ^2^)], *wR*(*F* ^2^), *S*	0.052, 0.145, 1.02
No. of reflections	5135
No. of parameters	330
H-atom treatment	H atoms treated by a mixture of independent and constrained refinement
Δρ_max_, Δρ_min_ (e Å^−3^)	0.19, −0.19

Computer programs: *APEX2*, *SAINT* and *XPREP* (Bruker, 2016 ▸), *SHELXT2018* (Sheldrick, 2015*a* ▸), *SHELXL2018* (Sheldrick, 2015*b* ▸), *ORTEP-3 for Windows* (Farrugia, 2012 ▸), *Mercury* (Macrae *et al.*, 2020 ▸) and *publCIF* (Westrip, 2010 ▸).

## supplementary crystallographic information

### Crystal data

**Table:**

C_31_H_35_NO_4_	*D*_x_ = 1.235 Mg m^−^^3^
*M**_r_* = 485.60	Melting point: 483 K
Monoclinic, *P*2_1_/*n*	Mo *K*α radiation, λ = 0.71073 Å
*a* = 10.4430 (6) Å	Cell parameters from 3522 reflections
*b* = 18.4563 (11) Å	θ = 2.4–21.4°
*c* = 14.2378 (9) Å	µ = 0.08 mm^−^^1^
β = 107.930 (2)°	*T* = 296 K
*V* = 2610.9 (3) Å^3^	BLOCK, yellow
*Z* = 4	0.40 × 0.30 × 0.20 mm
*F*(000) = 1040	

### Data collection

**Table:**

Bruker Kappa APEXII diffractometer	5135 independent reflections
Radiation source: fine-focus sealed tube	3061 reflections with *I* > 2σ(*I*)
Graphite monochromator	*R*_int_ = 0.096
ω and φ scan	θ_max_ = 26.0°, θ_min_ = 1.9°
Absorption correction: multi-scan (SADABS; Bruker, 2016)	*h* = −12→12
*T*_min_ = 0.953, *T*_max_ = 0.982	*k* = −22→22
37271 measured reflections	*l* = −17→17

### Refinement

**Table:**

Refinement on *F*^2^	Hydrogen site location: mixed
Least-squares matrix: full	H atoms treated by a mixture of independent and constrained refinement
*R*[*F*^2^ > 2σ(*F*^2^)] = 0.052	*w* = 1/[σ^2^(*F*_o_^2^) + (0.049*P*)^2^ + 1.0354*P*] where *P* = (*F*_o_^2^ + 2*F*_c_^2^)/3
*wR*(*F*^2^) = 0.145	(Δ/σ)_max_ < 0.001
*S* = 1.02	Δρ_max_ = 0.19 e Å^−^^3^
5135 reflections	Δρ_min_ = −0.19 e Å^−^^3^
330 parameters	Extinction correction: SHELXL-2018 (Sheldrick 2015b), Fc^*^=kFc[1+0.001xFc^2^λ^3^/sin(2θ)]^-1/4^
0 restraints	Extinction coefficient: 0.0095 (9)

### Special details

**Table:**

Geometry. All esds (except the esd in the dihedral angle between two l.s. planes) are estimated using the full covariance matrix. The cell esds are taken into account individually in the estimation of esds in distances, angles and torsion angles; correlations between esds in cell parameters are only used when they are defined by crystal symmetry. An approximate (isotropic) treatment of cell esds is used for estimating esds involving l.s. planes.

### Fractional atomic coordinates and isotropic or equivalent isotropic displacement parameters (Å^2^)

**Table:**

	*x*	*y*	*z*	*U*_iso_*/*U*_eq_	
C1	0.4603 (3)	0.09167 (14)	0.35227 (19)	0.0494 (7)	
H1	0.410397	0.089768	0.396205	0.059*	
C2	0.5467 (3)	0.03535 (15)	0.3501 (2)	0.0594 (8)	
H2	0.554683	−0.003955	0.392392	0.071*	
C3	0.6205 (3)	0.03758 (19)	0.2855 (3)	0.0730 (9)	
H3	0.678749	−0.000235	0.284067	0.088*	
C4	0.6087 (3)	0.0954 (2)	0.2230 (3)	0.0783 (10)	
H4	0.658479	0.096554	0.178917	0.094*	
C5	0.5225 (3)	0.15239 (16)	0.2254 (2)	0.0600 (8)	
H5	0.515644	0.191685	0.183241	0.072*	
C6	0.4467 (2)	0.15115 (13)	0.28985 (17)	0.0387 (6)	
C7	0.3509 (2)	0.21207 (12)	0.29182 (16)	0.0373 (6)	
H7A	0.363873	0.251425	0.250493	0.045*	
H7B	0.259084	0.194959	0.264697	0.045*	
C8	0.2902 (2)	0.21609 (11)	0.44966 (16)	0.0315 (5)	
C9	0.3279 (2)	0.22925 (11)	0.54880 (16)	0.0324 (5)	
C10	0.2338 (2)	0.21768 (12)	0.60510 (18)	0.0397 (6)	
C11	0.0907 (2)	0.19725 (14)	0.54753 (19)	0.0486 (7)	
H11A	0.040547	0.240759	0.520922	0.058*	
H11B	0.048053	0.174697	0.591747	0.058*	
C12	0.0856 (2)	0.14502 (13)	0.46279 (18)	0.0415 (6)	
C13	0.1588 (2)	0.17948 (13)	0.39549 (17)	0.0387 (6)	
H13A	0.176286	0.142145	0.353090	0.046*	
H13B	0.099539	0.214945	0.353460	0.046*	
C14	0.1542 (3)	0.07360 (14)	0.5055 (2)	0.0621 (8)	
H14A	0.151247	0.040740	0.452607	0.093*	
H14B	0.246283	0.082828	0.542975	0.093*	
H14C	0.108158	0.052524	0.547691	0.093*	
C15	−0.0603 (3)	0.12956 (17)	0.4002 (2)	0.0640 (8)	
H15A	−0.060687	0.096791	0.347760	0.096*	
H15B	−0.108169	0.108161	0.441002	0.096*	
H15C	−0.103353	0.174084	0.372739	0.096*	
C16	0.4694 (2)	0.25611 (11)	0.60194 (15)	0.0321 (5)	
H16	0.467180	0.283642	0.660269	0.039*	
C17	0.5082 (2)	0.30702 (11)	0.53197 (16)	0.0317 (5)	
C18	0.4708 (2)	0.29183 (11)	0.43328 (16)	0.0314 (5)	
C19	0.5302 (2)	0.33043 (12)	0.36254 (17)	0.0390 (6)	
H19A	0.460873	0.360347	0.318604	0.047*	
H19B	0.556907	0.294509	0.322496	0.047*	
C20	0.6522 (2)	0.37834 (13)	0.41290 (18)	0.0408 (6)	
C21	0.6196 (3)	0.42101 (13)	0.49464 (19)	0.0487 (7)	
H21A	0.696940	0.450341	0.528884	0.058*	
H21B	0.545178	0.453567	0.464992	0.058*	
C22	0.5830 (2)	0.37329 (12)	0.56887 (17)	0.0363 (5)	
C23	0.7793 (2)	0.33202 (16)	0.4554 (2)	0.0584 (8)	
H23A	0.798030	0.305676	0.403007	0.088*	
H23B	0.854105	0.362932	0.486968	0.088*	
H23C	0.765097	0.298526	0.502852	0.088*	
C24	0.6754 (3)	0.43065 (15)	0.3363 (2)	0.0599 (8)	
H24A	0.695827	0.403567	0.285106	0.090*	
H24B	0.595667	0.458964	0.308109	0.090*	
H24C	0.749294	0.462223	0.367536	0.090*	
C25	0.5679 (2)	0.19226 (11)	0.63605 (16)	0.0323 (5)	
C26	0.6453 (2)	0.16638 (12)	0.57974 (17)	0.0382 (6)	
H26	0.640648	0.189108	0.520478	0.046*	
C27	0.7300 (2)	0.10688 (12)	0.61051 (18)	0.0422 (6)	
H27	0.780002	0.090040	0.571065	0.051*	
C28	0.7407 (2)	0.07262 (12)	0.69872 (17)	0.0383 (6)	
C29	0.6655 (2)	0.09867 (12)	0.75739 (16)	0.0363 (5)	
C30	0.5783 (2)	0.15702 (12)	0.72569 (16)	0.0361 (5)	
H30	0.526356	0.172875	0.764243	0.043*	
C31	0.6164 (3)	0.08606 (17)	0.9100 (2)	0.0762 (10)	
H31A	0.639254	0.056029	0.967848	0.114*	
H31B	0.641437	0.135279	0.928749	0.114*	
H31C	0.521270	0.083510	0.877369	0.114*	
N1	0.37180 (17)	0.23959 (9)	0.39359 (13)	0.0323 (4)	
O1	0.61215 (17)	0.39158 (9)	0.65671 (12)	0.0508 (5)	
O2	0.26832 (18)	0.22551 (11)	0.69568 (13)	0.0585 (5)	
O3	0.82731 (18)	0.01471 (9)	0.72534 (15)	0.0533 (5)	
O4	0.68627 (18)	0.06155 (9)	0.84518 (13)	0.0554 (5)	
H3A	0.819 (3)	−0.009 (2)	0.782 (3)	0.113 (14)*	

### Atomic displacement parameters (Å^2^)

**Table:**

	*U*^11^	*U*^22^	*U*^33^	*U*^12^	*U*^13^	*U*^23^
C1	0.0509 (15)	0.0494 (16)	0.0490 (16)	0.0025 (13)	0.0172 (13)	−0.0031 (13)
C2	0.0593 (18)	0.0502 (17)	0.0631 (19)	0.0088 (14)	0.0105 (15)	−0.0073 (15)
C3	0.065 (2)	0.075 (2)	0.076 (2)	0.0198 (17)	0.0177 (18)	−0.0218 (19)
C4	0.074 (2)	0.100 (3)	0.075 (2)	0.011 (2)	0.0425 (18)	−0.017 (2)
C5	0.0650 (18)	0.072 (2)	0.0508 (17)	−0.0001 (16)	0.0284 (14)	−0.0038 (15)
C6	0.0358 (12)	0.0447 (14)	0.0335 (13)	−0.0070 (11)	0.0076 (10)	−0.0110 (11)
C7	0.0415 (13)	0.0401 (13)	0.0287 (12)	−0.0049 (11)	0.0087 (10)	−0.0021 (10)
C8	0.0337 (12)	0.0270 (11)	0.0333 (12)	0.0029 (9)	0.0093 (10)	0.0006 (10)
C9	0.0344 (12)	0.0309 (12)	0.0322 (12)	0.0036 (10)	0.0105 (10)	0.0020 (10)
C10	0.0458 (14)	0.0376 (13)	0.0374 (14)	0.0045 (11)	0.0155 (11)	0.0015 (11)
C11	0.0424 (14)	0.0537 (16)	0.0537 (16)	−0.0016 (12)	0.0206 (12)	−0.0004 (13)
C12	0.0395 (13)	0.0406 (14)	0.0444 (14)	−0.0046 (11)	0.0130 (11)	0.0014 (11)
C13	0.0370 (13)	0.0393 (13)	0.0376 (13)	−0.0032 (11)	0.0083 (10)	0.0000 (11)
C14	0.078 (2)	0.0404 (15)	0.068 (2)	0.0007 (14)	0.0221 (16)	0.0101 (14)
C15	0.0470 (16)	0.078 (2)	0.0665 (19)	−0.0225 (15)	0.0166 (14)	−0.0063 (16)
C16	0.0373 (12)	0.0299 (12)	0.0276 (11)	0.0019 (10)	0.0078 (9)	−0.0025 (9)
C17	0.0331 (12)	0.0281 (11)	0.0320 (12)	0.0007 (9)	0.0071 (10)	−0.0003 (10)
C18	0.0306 (11)	0.0278 (11)	0.0335 (12)	0.0014 (9)	0.0065 (9)	0.0018 (10)
C19	0.0423 (13)	0.0375 (13)	0.0369 (13)	−0.0016 (11)	0.0118 (11)	0.0041 (11)
C20	0.0435 (14)	0.0367 (13)	0.0441 (14)	−0.0068 (11)	0.0163 (11)	0.0004 (11)
C21	0.0626 (17)	0.0347 (13)	0.0523 (16)	−0.0116 (12)	0.0228 (13)	−0.0058 (12)
C22	0.0352 (12)	0.0318 (12)	0.0390 (14)	0.0019 (10)	0.0071 (10)	−0.0030 (11)
C23	0.0392 (14)	0.0672 (19)	0.0686 (19)	0.0025 (13)	0.0164 (13)	0.0030 (16)
C24	0.0667 (18)	0.0572 (17)	0.0613 (19)	−0.0174 (14)	0.0277 (15)	0.0053 (14)
C25	0.0310 (12)	0.0289 (12)	0.0328 (12)	−0.0031 (9)	0.0035 (10)	−0.0020 (10)
C26	0.0447 (14)	0.0349 (13)	0.0344 (13)	0.0041 (11)	0.0110 (11)	0.0050 (10)
C27	0.0467 (14)	0.0387 (14)	0.0445 (15)	0.0076 (11)	0.0188 (12)	0.0031 (12)
C28	0.0379 (13)	0.0298 (12)	0.0425 (14)	0.0028 (10)	0.0055 (11)	0.0017 (11)
C29	0.0429 (13)	0.0327 (12)	0.0309 (12)	−0.0008 (10)	0.0078 (10)	0.0066 (10)
C30	0.0413 (13)	0.0328 (12)	0.0336 (13)	−0.0001 (10)	0.0106 (10)	−0.0014 (10)
C31	0.117 (3)	0.072 (2)	0.0530 (18)	0.0312 (19)	0.0444 (19)	0.0244 (16)
N1	0.0347 (10)	0.0340 (10)	0.0270 (10)	−0.0035 (8)	0.0078 (8)	−0.0013 (8)
O1	0.0639 (12)	0.0427 (10)	0.0406 (10)	−0.0104 (8)	0.0084 (8)	−0.0098 (8)
O2	0.0616 (12)	0.0802 (14)	0.0377 (10)	−0.0048 (10)	0.0213 (9)	−0.0042 (10)
O3	0.0598 (12)	0.0433 (10)	0.0583 (12)	0.0200 (9)	0.0205 (10)	0.0155 (9)
O4	0.0753 (13)	0.0514 (11)	0.0431 (10)	0.0193 (9)	0.0236 (9)	0.0164 (9)

### Geometric parameters (Å, º)

**Table:**

C1—C2	1.383 (3)	C16—H16	0.9800
C1—C6	1.392 (3)	C17—C18	1.367 (3)
C1—H1	0.9300	C17—C22	1.459 (3)
C2—C3	1.371 (4)	C18—N1	1.400 (3)
C2—H2	0.9300	C18—C19	1.513 (3)
C3—C4	1.370 (5)	C19—C20	1.535 (3)
C3—H3	0.9300	C19—H19A	0.9700
C4—C5	1.392 (4)	C19—H19B	0.9700
C4—H4	0.9300	C20—C21	1.528 (3)
C5—C6	1.384 (3)	C20—C24	1.531 (3)
C5—H5	0.9300	C20—C23	1.538 (3)
C6—C7	1.511 (3)	C21—C22	1.512 (3)
C7—N1	1.487 (3)	C21—H21A	0.9700
C7—H7A	0.9700	C21—H21B	0.9700
C7—H7B	0.9700	C22—O1	1.240 (3)
C8—C9	1.365 (3)	C23—H23A	0.9600
C8—N1	1.404 (3)	C23—H23B	0.9600
C8—C13	1.511 (3)	C23—H23C	0.9600
C9—C10	1.462 (3)	C24—H24A	0.9600
C9—C16	1.522 (3)	C24—H24B	0.9600
C10—O2	1.236 (3)	C24—H24C	0.9600
C10—C11	1.514 (3)	C25—C26	1.387 (3)
C11—C12	1.532 (3)	C25—C30	1.407 (3)
C11—H11A	0.9700	C26—C27	1.393 (3)
C11—H11B	0.9700	C26—H26	0.9300
C12—C14	1.534 (3)	C27—C28	1.380 (3)
C12—C13	1.535 (3)	C27—H27	0.9300
C12—C15	1.537 (3)	C28—O3	1.376 (3)
C13—H13A	0.9700	C28—C29	1.396 (3)
C13—H13B	0.9700	C29—O4	1.383 (3)
C14—H14A	0.9600	C29—C30	1.393 (3)
C14—H14B	0.9600	C30—H30	0.9300
C14—H14C	0.9600	C31—O4	1.415 (3)
C15—H15A	0.9600	C31—H31A	0.9600
C15—H15B	0.9600	C31—H31B	0.9600
C15—H15C	0.9600	C31—H31C	0.9600
C16—C17	1.512 (3)	O3—H3A	0.94 (4)
C16—C25	1.541 (3)		
			
C2—C1—C6	121.2 (3)	C18—C17—C22	119.6 (2)
C2—C1—H1	119.4	C18—C17—C16	120.02 (19)
C6—C1—H1	119.4	C22—C17—C16	120.38 (19)
C3—C2—C1	119.9 (3)	C17—C18—N1	119.9 (2)
C3—C2—H2	120.1	C17—C18—C19	122.82 (19)
C1—C2—H2	120.1	N1—C18—C19	117.30 (18)
C4—C3—C2	120.2 (3)	C18—C19—C20	114.28 (18)
C4—C3—H3	119.9	C18—C19—H19A	108.7
C2—C3—H3	119.9	C20—C19—H19A	108.7
C3—C4—C5	120.1 (3)	C18—C19—H19B	108.7
C3—C4—H4	119.9	C20—C19—H19B	108.7
C5—C4—H4	119.9	H19A—C19—H19B	107.6
C6—C5—C4	120.7 (3)	C21—C20—C24	109.8 (2)
C6—C5—H5	119.7	C21—C20—C19	107.77 (19)
C4—C5—H5	119.7	C24—C20—C19	108.7 (2)
C5—C6—C1	118.0 (2)	C21—C20—C23	110.8 (2)
C5—C6—C7	121.1 (2)	C24—C20—C23	108.9 (2)
C1—C6—C7	120.9 (2)	C19—C20—C23	110.8 (2)
N1—C7—C6	111.90 (18)	C22—C21—C20	113.29 (19)
N1—C7—H7A	109.2	C22—C21—H21A	108.9
C6—C7—H7A	109.2	C20—C21—H21A	108.9
N1—C7—H7B	109.2	C22—C21—H21B	108.9
C6—C7—H7B	109.2	C20—C21—H21B	108.9
H7A—C7—H7B	107.9	H21A—C21—H21B	107.7
C9—C8—N1	120.03 (19)	O1—C22—C17	122.0 (2)
C9—C8—C13	122.3 (2)	O1—C22—C21	120.7 (2)
N1—C8—C13	117.57 (19)	C17—C22—C21	117.3 (2)
C8—C9—C10	120.8 (2)	C20—C23—H23A	109.5
C8—C9—C16	119.9 (2)	C20—C23—H23B	109.5
C10—C9—C16	119.26 (19)	H23A—C23—H23B	109.5
O2—C10—C9	121.7 (2)	C20—C23—H23C	109.5
O2—C10—C11	121.1 (2)	H23A—C23—H23C	109.5
C9—C10—C11	117.2 (2)	H23B—C23—H23C	109.5
C10—C11—C12	111.8 (2)	C20—C24—H24A	109.5
C10—C11—H11A	109.2	C20—C24—H24B	109.5
C12—C11—H11A	109.2	H24A—C24—H24B	109.5
C10—C11—H11B	109.2	C20—C24—H24C	109.5
C12—C11—H11B	109.2	H24A—C24—H24C	109.5
H11A—C11—H11B	107.9	H24B—C24—H24C	109.5
C11—C12—C14	109.2 (2)	C26—C25—C30	118.0 (2)
C11—C12—C13	109.12 (19)	C26—C25—C16	122.2 (2)
C14—C12—C13	110.0 (2)	C30—C25—C16	119.8 (2)
C11—C12—C15	111.2 (2)	C25—C26—C27	121.1 (2)
C14—C12—C15	109.2 (2)	C25—C26—H26	119.5
C13—C12—C15	108.1 (2)	C27—C26—H26	119.5
C8—C13—C12	114.50 (19)	C28—C27—C26	120.9 (2)
C8—C13—H13A	108.6	C28—C27—H27	119.6
C12—C13—H13A	108.6	C26—C27—H27	119.6
C8—C13—H13B	108.6	O3—C28—C27	118.0 (2)
C12—C13—H13B	108.6	O3—C28—C29	123.1 (2)
H13A—C13—H13B	107.6	C27—C28—C29	119.0 (2)
C12—C14—H14A	109.5	O4—C29—C30	125.5 (2)
C12—C14—H14B	109.5	O4—C29—C28	114.2 (2)
H14A—C14—H14B	109.5	C30—C29—C28	120.3 (2)
C12—C14—H14C	109.5	C29—C30—C25	120.8 (2)
H14A—C14—H14C	109.5	C29—C30—H30	119.6
H14B—C14—H14C	109.5	C25—C30—H30	119.6
C12—C15—H15A	109.5	O4—C31—H31A	109.5
C12—C15—H15B	109.5	O4—C31—H31B	109.5
H15A—C15—H15B	109.5	H31A—C31—H31B	109.5
C12—C15—H15C	109.5	O4—C31—H31C	109.5
H15A—C15—H15C	109.5	H31A—C31—H31C	109.5
H15B—C15—H15C	109.5	H31B—C31—H31C	109.5
C17—C16—C9	106.92 (17)	C18—N1—C8	119.06 (18)
C17—C16—C25	113.24 (18)	C18—N1—C7	119.81 (18)
C9—C16—C25	111.08 (17)	C8—N1—C7	121.08 (17)
C17—C16—H16	108.5	C28—O3—H3A	112 (2)
C9—C16—H16	108.5	C29—O4—C31	117.53 (19)
C25—C16—H16	108.5		
			
C6—C1—C2—C3	−0.1 (4)	C18—C19—C20—C21	−44.2 (3)
C1—C2—C3—C4	−0.1 (5)	C18—C19—C20—C24	−163.2 (2)
C2—C3—C4—C5	0.5 (5)	C18—C19—C20—C23	77.2 (3)
C3—C4—C5—C6	−0.7 (5)	C24—C20—C21—C22	174.9 (2)
C4—C5—C6—C1	0.4 (4)	C19—C20—C21—C22	56.6 (3)
C4—C5—C6—C7	−178.9 (2)	C23—C20—C21—C22	−64.8 (3)
C2—C1—C6—C5	−0.1 (4)	C18—C17—C22—O1	175.6 (2)
C2—C1—C6—C7	179.3 (2)	C16—C17—C22—O1	−3.1 (3)
C5—C6—C7—N1	−129.5 (2)	C18—C17—C22—C21	−2.0 (3)
C1—C6—C7—N1	51.1 (3)	C16—C17—C22—C21	179.3 (2)
N1—C8—C9—C10	168.45 (19)	C20—C21—C22—O1	147.2 (2)
C13—C8—C9—C10	−8.8 (3)	C20—C21—C22—C17	−35.1 (3)
N1—C8—C9—C16	−11.7 (3)	C17—C16—C25—C26	−26.1 (3)
C13—C8—C9—C16	171.10 (19)	C9—C16—C25—C26	94.3 (2)
C8—C9—C10—O2	176.0 (2)	C17—C16—C25—C30	155.81 (19)
C16—C9—C10—O2	−3.9 (3)	C9—C16—C25—C30	−83.9 (2)
C8—C9—C10—C11	−5.3 (3)	C30—C25—C26—C27	0.5 (3)
C16—C9—C10—C11	174.81 (19)	C16—C25—C26—C27	−177.6 (2)
O2—C10—C11—C12	−142.6 (2)	C25—C26—C27—C28	−0.9 (3)
C9—C10—C11—C12	38.8 (3)	C26—C27—C28—O3	−179.3 (2)
C10—C11—C12—C14	63.9 (3)	C26—C27—C28—C29	−0.3 (3)
C10—C11—C12—C13	−56.4 (3)	O3—C28—C29—O4	0.5 (3)
C10—C11—C12—C15	−175.5 (2)	C27—C28—C29—O4	−178.5 (2)
C9—C8—C13—C12	−11.7 (3)	O3—C28—C29—C30	−179.2 (2)
N1—C8—C13—C12	171.05 (19)	C27—C28—C29—C30	1.8 (3)
C11—C12—C13—C8	43.4 (3)	O4—C29—C30—C25	178.1 (2)
C14—C12—C13—C8	−76.5 (3)	C28—C29—C30—C25	−2.2 (3)
C15—C12—C13—C8	164.4 (2)	C26—C25—C30—C29	1.0 (3)
C8—C9—C16—C17	36.7 (3)	C16—C25—C30—C29	179.24 (19)
C10—C9—C16—C17	−143.48 (19)	C17—C18—N1—C8	15.0 (3)
C8—C9—C16—C25	−87.3 (2)	C19—C18—N1—C8	−163.75 (19)
C10—C9—C16—C25	92.5 (2)	C17—C18—N1—C7	−167.41 (19)
C9—C16—C17—C18	−38.3 (3)	C19—C18—N1—C7	13.8 (3)
C25—C16—C17—C18	84.3 (2)	C9—C8—N1—C18	−16.6 (3)
C9—C16—C17—C22	140.35 (19)	C13—C8—N1—C18	160.74 (18)
C25—C16—C17—C22	−97.0 (2)	C9—C8—N1—C7	165.86 (19)
C22—C17—C18—N1	−163.77 (18)	C13—C8—N1—C7	−16.8 (3)
C16—C17—C18—N1	14.9 (3)	C6—C7—N1—C18	84.6 (2)
C22—C17—C18—C19	14.9 (3)	C6—C7—N1—C8	−97.9 (2)
C16—C17—C18—C19	−166.38 (19)	C30—C29—O4—C31	−2.1 (4)
C17—C18—C19—C20	10.0 (3)	C28—C29—O4—C31	178.3 (2)
N1—C18—C19—C20	−171.28 (19)		

### Hydrogen-bond geometry (Å, º)

**Table:**

*D*—H···*A*	*D*—H	H···*A*	*D*···*A*	*D*—H···*A*
O3—H3*A*···O1^i^	0.94 (4)	2.07 (4)	2.780 (2)	131 (3)
C7—H7*B*···O1^ii^	0.97	2.41	3.260 (3)	146

Symmetry codes: (i) −*x*+3/2, *y*−1/2, −*z*+3/2; (ii) *x*−1/2, −*y*+1/2, *z*−1/2.
